# Influence of Dual Heat Treatment on the Metallurgy and Mechanical Behaviour of Diamond‐Like Carbon–Coated NiTi Rotary Systems: A Multimethod Investigation

**DOI:** 10.1111/iej.70089

**Published:** 2025-12-30

**Authors:** Emmanuel João Nogueira Leal Silva, Thyago Oliveira Cardoso, Jorge N. R. Martins, Francisco Manuel Braz Fernandes, Victor Talarico Leal Vieira, Marco A. Versiani

**Affiliations:** ^1^ Grande Rio University (UNIGRANRIO) Rio de Janeiro Brazil; ^2^ Rio de Janeiro State University (UERJ) Rio de Janeiro Brazil; ^3^ Faculdade de Medicina Dentária Universidade de Lisboa Lisboa Portugal; ^4^ Grupo de Investigação Em Bioquimica e Biologia Oral, Unidade de Investigação Em Ciências Orais e Biomédicas (UICOB), Faculdade de Medicina Dentária Universidade de Lisboa Lisboa Portugal; ^5^ Centro de Estudo de Medicina Dentária Baseada Na Evidência (CEMDBE) ‐ Cochrane Portugal, Faculdade de Medicina Dentária Universidade de Lisboa Lisboa Portugal; ^6^ LIBPhys‐FCT UID/FIS/04559/2013, Faculdade de Medicina Dentária Universidade de Lisboa Lisboa Portugal; ^7^ CENIMAT/I3N, Department of Materials Science, NOVA School of Science and Technology Universidade NOVA de Lisboa Caparica Portugal; ^8^ Dental Specialty Center, Brazilian Military Police Minas Gerais Brazil

**Keywords:** endodontics, heat treatment, mechanical properties, metallurgical properties, nickel–titanium instruments

## Abstract

**Aim:**

To investigate how the application of dual heat treatment during the manufacturing process influences the mechanical performance (flexibility, cyclic fatigue resistance, torsional strength, bending, buckling and cutting efficiency) of rotary nickel‐titanium endodontic instruments.

**Methodology:**

Rainbow One (*n* = 58) and Rainbow Ultra (*n* = 58) instruments (size 25/0.06, 25 mm length) were evaluated. Geometric analysis was performed using optical 3D scanning and SEM to assess cutting edges, tip design and surface features. Metallurgical characterisation included EDS for elemental composition and DSC for phase transformation temperatures, with segments obtained from the tip, middle and coronal blade regions. Mechanical testing comprised cyclic fatigue, torsional strength (torque to failure, maximum angle of rotation), bending resistance, buckling strength and cutting efficiency. Standardised testing protocols were applied, including ASTM and ISO guidelines for mechanical assessments. Statistical analyses were conducted using Shapiro–Wilk and Levene tests to assess assumptions, followed by independent Student's *t*‐tests, with significance set at 5%.

**Results:**

Both instruments shared equivalent geometries, with deviations below 100 μm, and similar tip designs and surface characteristics. EDS confirmed near‐equiatomic NiTi composition for both systems. DSC revealed R‐phase crystallographic structure at room temperature for both instruments; however, Rainbow Ultra exhibited higher phase transformation temperatures (Rs 35°C vs. 25°C; Rf 45°C vs. 31°C; As 30°C vs. 24°C; Af 50°C vs. 38°C) and a double austenitic transformation peak, compared with a single peak for Rainbow One. Mechanically, Rainbow Ultra demonstrated greater cyclic fatigue resistance (95.5° ± 7 vs. 79.1 ± 6 s), higher maximum rotation angle (485° ± 49° vs. 402° ± 50°), and improved cutting efficiency (*p* < 0.05). Rainbow One exhibited higher torque to failure (1.8° ± 0.2 vs. 1.4° ± 0.1 N·cm), greater bending load (369 ± 34 vs. 336 ± 14 gf), and higher buckling resistance (339 ± 32 vs. 273 ± 24 gf), indicating stiffer behaviour.

**Conclusions:**

The application of dual heat treatment during manufacturing significantly influenced the mechanical performance of the tested rotary NiTi instruments. Rainbow Ultra, subjected to sequential heat treatments, exhibited higher flexibility, cyclic fatigue resistance and cutting efficiency, whereas Rainbow One demonstrated greater torsional strength and buckling resistance.

## 1 Introduction

The introduction of nickel–titanium (NiTi) rotary instruments marked a major milestone in modern endodontics, transforming root canal shaping procedures and improving clinical outcomes (Bürklein and Arias 2022). Their unique properties, including super elasticity and thermally induced shape memory, confer enhanced flexibility, allowing adaptation to complex canal curvatures and reducing the incidence of procedural errors such as transportation, ledges and perforations (Shen et al. 2013; Zupanc et al. 2018; Bürklein and Arias 2022; Barbosa et al. 2025). Advances in metallurgy and instrument design have further diversified available systems, which now feature distinct kinematics, heat treatments and geometries aimed at improving safety, efficiency and clinical predictability (Zupanc et al. 2018; Silva et al. 2020; Martins et al. 2022).

Although the overall risk of instrument separation has been substantially reduced when procedures are performed by adequately trained clinicians (Plotino et al. 2015; Rodrigues et al. 2016; Bueno et al. 2020), it is important to recognise that the majority of root canal treatments worldwide are carried out by general practitioners (Savani et al. 2014; Mungia et al. 2025; Nosrat et al. 2025), whose level of endodontic training may vary considerably. Consequently, the risk of instrument separation has not been completely eliminated and remains a clinically relevant concern, particularly in curved or calcified canals, where it can potentially compromise treatment outcomes and favour persistent apical infection (Ng et al. 2011; McGuigan et al. 2013). To address this, manufacturers have developed novel heat treatment processes that modify the alloy's microstructure, typically through controlled heating followed by gradual cooling, resulting in a predominance of the martensitic phase at room temperature. These modifications enhance flexibility and cyclic fatigue resistance, thereby reducing the risk of fracture during clinical use (Shen et al. 2013; Zupanc et al. 2018; Martins et al. 2022). Consequently, a variety of NiTi instruments with proprietary heat treatments are now available, presenting a broad range of mechanical and metallurgical characteristics. While this diversity increases clinical options, it also requires careful evaluation of how variations in metallurgy and thermal processing affect instrument behaviour and performance.

The Rainbow One system (Ramo Medical, Suzhou, China) is a heat‐treated NiTi rotary system (subjected to post‐machining heat treatment) with an S‐shaped cross‐section and a multi‐coloured diamond‐like carbon (DLC) surface coating. Produced via ion deposition in a vacuum furnace, the coating generates the characteristic colour gradient and is designed to enhance surface hardness and chemical resistance, potentially improving durability (Roy and Lee 2007; Malisz et al. 2023). A recent multimethod evaluation of the Rainbow One system demonstrated a balanced mechanical profile; however, compared with other heat‐treated rotary systems, it exhibited lower flexibility, smaller deflection angles and reduced cutting efficiency, suggesting stiffer behaviour and potentially limited adaptability in sharply curved canals (Silva, Martins, Ajuz, et al. 2024). A new version, the Rainbow Ultra system (Ramo Medical), was recently introduced, offering improved flexibility and cutting efficiency while retaining the same geometric design of Rainbow One instruments (https://bit.ly/43NDdy0). According to the manufacturer, Rainbow Ultra undergoes two sequential heat treatment cycles: one prior to machining and another post‐machining, followed by DLC coating. The initial pre‐machining treatment softens the alloy, facilitating cutting and minimising surface defects generated during grinding. The post‐machining treatment relieves internal stresses, restores the desired phase composition and refines the microstructure, thereby enhancing flexibility, fatigue resistance and overall consistency. Therefore, the present study aimed to investigate how the application of dual heat treatment during the manufacturing process influences the mechanical performance (cyclic fatigue resistance, torsional strength, bending resistance, buckling resistance and cutting efficiency) of rotary NiTi endodontic instruments. The null hypothesis was that no significant differences exist between Rainbow One and Rainbow Ultra instruments for the tested parameters.

## 2 Materials and Methods

This manuscript is reported in accordance with the Preferred Reporting Items for Laboratory Studies in Endodontology (PRILE) (Figure S1).

### 2.1 Sample Selection

**FIGURE 1:**
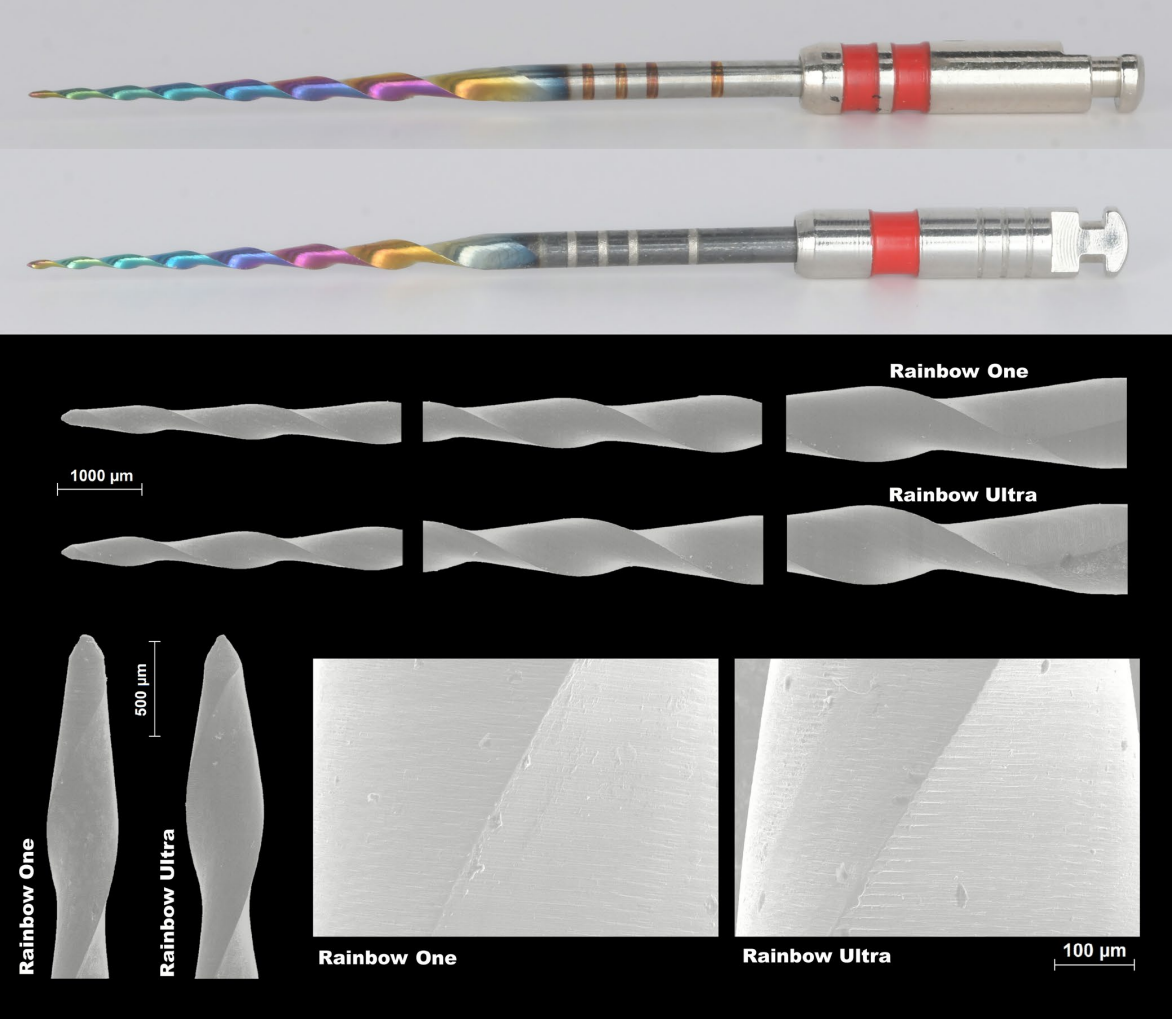
Representative macroscopic images of the tested instruments (top) showing the characteristic black coloration in the alloy regions of Rainbow Ultra not covered by the DLC coating. Corresponding scanning electron microscopy images (bottom) illustrate the cutting edges, tips, and surfaces, demonstrating equivalent designs and surface characteristics for both instruments.

Rainbow One (*n* = 58) and Rainbow Ultra (*n* = 58) instruments (Ramo Medical, Suzhou, China) were selected for testing. Both had a tip size of 25 and a constant 0.06 taper, a length of 25 mm, and similar geometries, differing only in their heat treatment (Figure 1). Prior to testing, all instruments were examined under a microscope at 13.6× magnification (Opmi Pico; Carl Zeiss Surgical) to identify any irregularities in the cutting blades or signs of unwinding that might compromise structural integrity. No defects were detected and all instruments met the inclusion criteria.

### 2.2 Design Analysis

One instrument from each group was randomly selected and scanned using an optical 3D measurement system (ATOS Q, GOM, Braunschweig, Germany) equipped with an ATOS Capsule 12 M MV40 sensor (GOM, Braunschweig, Germany). Before scanning, a uniform anti‐reflective coating (~2 μm thick) was applied to the active surface, and the instruments were securely mounted in an object holder. Image acquisition was performed using 12‐megapixel resolution in STL format from 24 distinct orientations, providing a complete 360° view. The working distance was fixed at 290 mm, and the measurement volume was calibrated to 40 × 40 × 40 mm. No modifications or smoothing were applied to the raw meshes. The STL files from each instrument were superimposed using dedicated software (Geomagic Control X; 3D Systems, Cary, NC, USA), and geometric deviations were analysed with threshold values of 100 and 1000 μm. Additionally, three randomly selected instruments from each group were examined under a scanning electron microscope (SEM) (S‐2400, Hitachi, Tokyo, Japan) to assess the shape of the cutting edges, the tip design (active or non‐active), and surface features related to machining marks or differences in polishing procedures.

### 2.3 Metallurgical Characteristics

The metallurgical characteristics of the samples were evaluated using energy‐dispersive X‐ray spectroscopy (EDS) and differential scanning calorimetry (DSC). Semi‐quantitative elemental analysis was performed on three instruments per group using a SEM system (DSM‐962, Carl Zeiss Microscopy GmbH, Jena, Germany) equipped with an Inca X‐act EDS detector (Oxford Instruments NanoAnalysis, Abingdon, UK). The system operated at 20 kV accelerating voltage and 3.1 A beam current, following a 10 min vacuum preparation. Spectra were collected over a 500 × 500 μm area for 1 min at a working distance of 25 mm. The ZAF correction method was applied, and processed data were analysed using Microanalysis Suite software v4.14 (Oxford Instruments NanoAnalysis, Abingdon, UK) to determine metallic element concentrations.

DSC analysis was conducted (ASTM International 2004) using a DSC 204 F1 Phoenix system (Netzsch‐Gerätebau GmbH, Selb, Germany). Segments 3–5 mm in length were obtained from the apical, middle, and coronal regions of the active blade. Each segment was chemically etched (25% hydrofluoric acid, 45% nitric acid, 30% distilled water) for approximately 2 min, then rinsed with distilled water. Thermal cycling involved equilibration at room temperature for 2 min, heating to 150°C at 10°C/min with a 2 min isothermal hold, cooling to −150°C at the same rate with another 2 min hold, and reheating to 150°C under identical conditions before final cooling to room temperature. Data were analysed using Proteus Thermal Analysis software (Netzsch‐Gerätebau GmbH), and phase transformation temperatures were determined by the tangent method.

### 2.4 Mechanical Performance

Six mechanical parameters were evaluated: time to fracture, torque to failure, maximum angle to fracture, bending strength, buckling strength, and cutting efficiency. Sample size was estimated based on preliminary tests (*n* = 5), using a significance level of 0.05, statistical power of 80%, and the following effect sizes: 3.85 (fracture time), 7.33 (torque to failure), 1.64 (angle to fracture), 3.07 (bending strength), 1.38 (buckling strength) and 3.35 (cutting efficiency). The calculated minimum sample sizes were 3, 2, 6, 3, 8 and 3, respectively. To ensure statistical robustness, 10 instruments were tested for each parameter.

The cyclic fatigue test was conducted according to the guidelines of the American Society of Testing Materials (Peters et al. 2020) and a new fixture outlined in a proposal for an updated ISO 3630‐1 standard (ISO 3630‐3631 2008). The instruments were adapted to a 6:1 reduction handpiece (Sirona Dental Systems GmbH, Bensheim, Germany) and connected to a torque‐controlled motor (VDW Silver; VDW GmbH) operating in rotary motion at 400 rpm and 2.0 N torque. Instruments were statically activated within a stainless‐steel curved tube apparatus (radius of 6 mm and angle of 86°). The fracture moment was identified through visual and auditory examination, and the time to fracture (in seconds) was recorded using a digital chronometer. Torsional strength (maximum torque [N.cm] and angle of rotation [°]) and bending resistance (maximum bending load [gf]) tests were conducted following international standard guidelines (ISO 3630‐3631 2008). The highest buckling load (N) was determined using a universal testing machine with a 1 kN load cell (Instron Corporation 4502; series H3307) (Martins et al. 2022; Silva et al. 2025). Cutting efficiency was assessed using a custom apparatus linking an endodontic motor (VDW Gold) to a 500 N load cell. Each instrument was positioned at the uppermost part of a simulated straight canal (size 15/0.02) within a bone block model (PCF 10; Sawbones, Vashon, WA, USA), pre‐loaded with 10 gf by the universal testing machine, and then operated at 400 rpm and 2.0 N torque, advancing 3 mm forward and 2 mm backward per cycle, resulting in a net progression of 1 mm per cycle. Maximum force recorded during the test indicated cutting efficiency, with higher force implying lower cutting ability.

### 2.5 Statistical Analysis

The Shapiro–Wilk and Levene tests were applied to assess the assumptions of normality and homogeneity of variances, respectively. Subsequently, group comparisons were performed using independent Student's *t*‐tests. The significance level for all analyses was set at 5% (SPSS v25.0 for Windows; SPSS Inc., Chicago, IL, USA).

## 3 Results

### 3.1 Design Analysis

**FIGURE 2:**
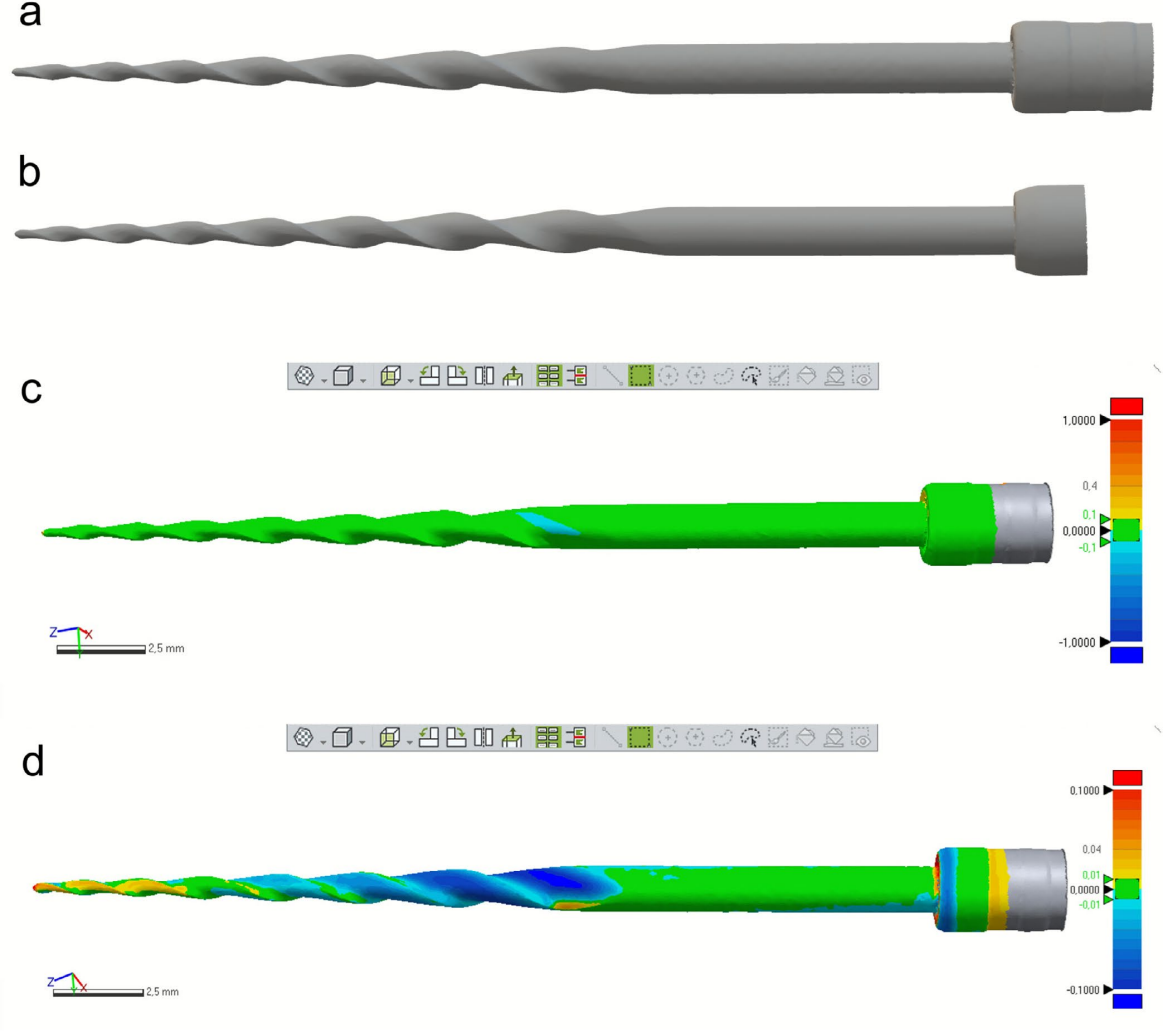
High‐resolution optical 3D models of Rainbow One (a) and Rainbow Ultra (b) shown at the top, illustrating highly similar designs. The bottom panels show the superimposition of the 3D models, with most deviations below 100 μm using a 1000 μm threshold (c) and the majority within the ±40 μm range using a 100 μm threshold (d).

Macroscopically, the primary difference between the Rainbow One and Rainbow Ultra instruments was the black coloration of the latter in the alloy regions not covered by the multicoloured DLC coating (Figure 1). Superimposition of the STL meshes confirmed that both instruments shared equivalent geometries (Figure 2a,b), with most deviations below 100 μm (Figure 2c) and predominantly within the ±40 μm range (Figure 2d). SEM analysis showed similar cutting edge and tip designs. Both instruments exhibited fine, parallel machining marks of comparable intensity, with no discernible differences in surface finishing or polishing quality (Figure 1).

### 3.2 Metallurgical Characteristics

**FIGURE 3:**
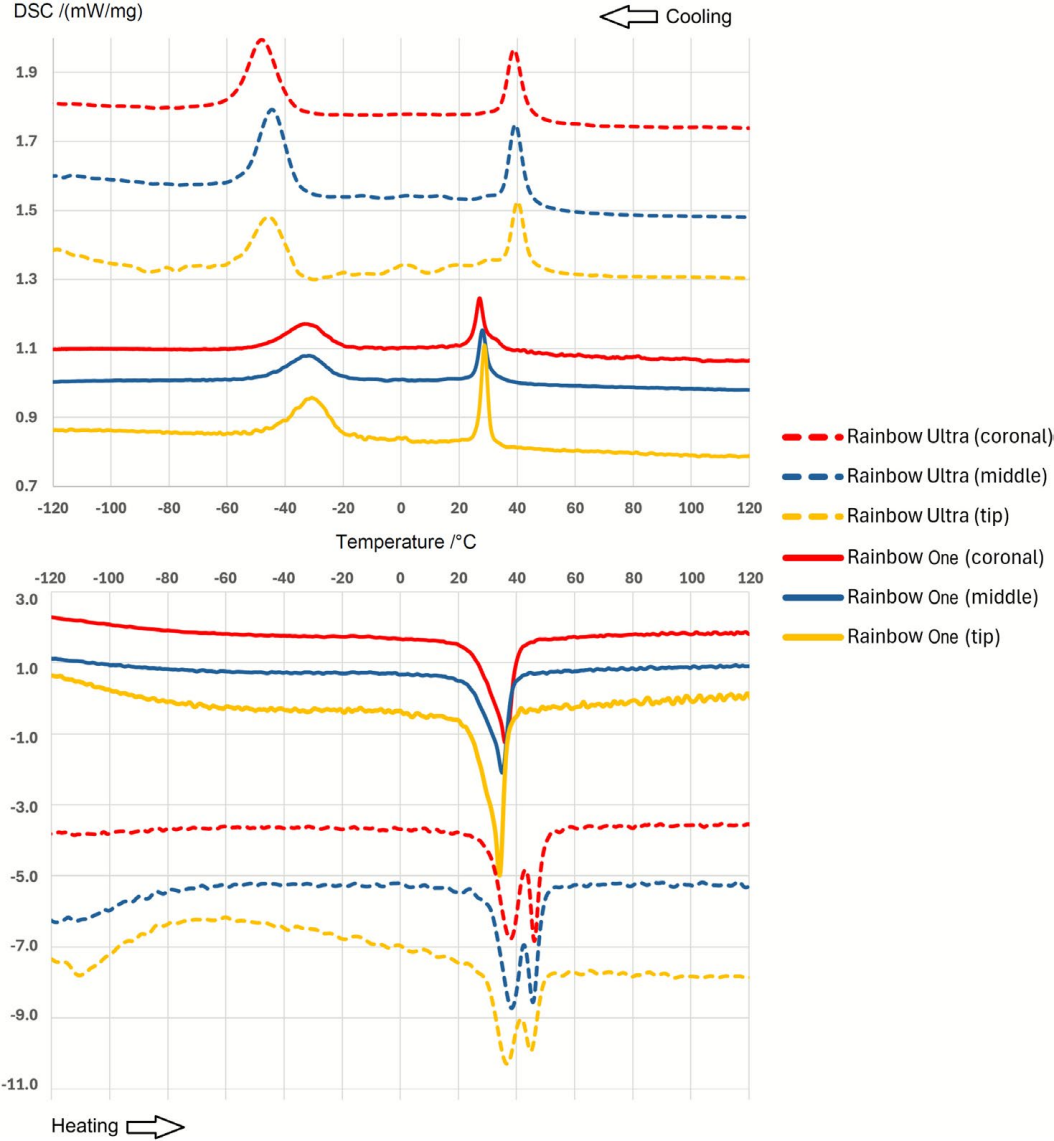
Differential scanning calorimetry (DSC) curves depicting the phase transformation temperatures of Rainbow One and Rainbow Ultra instruments. The top panel shows the cooling curves (read from right to left), and the bottom panel shows the heating curves (read from left to right). Both instruments exhibit an R‐phase crystallographic structure at room temperature; however, Rainbow Ultra demonstrates higher phase transformation temperatures than Rainbow One. As the temperature rises from room temperature toward body temperature, Rainbow One transitions to the austenitic phase, whereas Rainbow Ultra predominantly retains its R‐phase structure.

EDS analysis confirmed that both Rainbow One and Rainbow Ultra instruments were composed of near‐equiatomic NiTi alloy, with no detectable traces of other metallic elements (Table 1). In the DSC analysis, the three segments obtained from each instrument (tip, middle and coronal blade) exhibited consistent thermal behaviour within their respective groups. However, notable differences were observed between the Rainbow One and Rainbow Ultra groups (Table 1, Figure 3). Overall, both instruments displayed an R‐phase crystallographic structure at room temperature, as determined by the tangent method, but the phase transformation temperatures were higher for Rainbow Ultra. Rainbow One instruments exhibited R‐phase start (Rs) and finish (Rf) temperatures of approximately 25°C and 31°C, respectively, whereas Rainbow Ultra showed values around 35°C and 45°C. The austenite start (As) and finish (Af) temperatures were also higher for Rainbow Ultra (~30°C and 50°C) compared with Rainbow One (~24°C and 38°C). Additionally, while the austenitic transformation peak appeared as a single peak in Rainbow One, it was observed as a double peak in Rainbow Ultra instruments (Table 1, Figure 3).

**TABLE 1:** Metallurgical characteristics of both Rainbow and Rainbow Ultra.

Instrument/sample	DSC (°C)						EDS (ratio NiTi)
	Cooling				Heating		
	Rs	Rf	Ms	Mf	As	Af	
Rainbow One (coronal)	31.3	24.2	−19.7	−48.9	24.6	40.4	1.013
Rainbow One (middle)	31.7	24.8	−20.5	−47.3	23.7	38.9	1.041
Rainbow One (tip)	31.2	25.6	−20.1	−44.6	24.0	37.6	1.023
Rainbow Ultra (coronal)	44.7	34.3	−37.5	−57.6	30.5	49.4	1.035
Rainbow Ultra (middle)	45.0	35.0	−35.2	−54.0	30.4	49.4	1.018
Rainbow Ultra (tip)	45.9	36.1	−34.7	−56.2	29.3	50.8	1.054

Abbreviations: Rs R‐phase start; Rf R‐phase finish; Ms. Martensite start; Mf Martensite finish; As Austenite start; Af Austenite finish; DSC Differential scanning calorimetry; EDS Energy‐dispersive X‐ray spectroscopy.

### 3.3 Mechanical Performance

Rainbow Ultra exhibited significantly greater cyclic fatigue resistance (95.5 ± 7 s) than Rainbow One (79.1 ± 6 s) (*p* < 0.0001). In contrast, Rainbow One showed higher torque to failure (1.8 ± 0.2 N·cm) than Rainbow Ultra (1.4 ± 0.1 N·cm) (*p* = 0.0014). The maximum angle of rotation was also greater for Rainbow Ultra (485°± 49°) compared with Rainbow One (402° ± 50°) (*p* = 0.0016). Regarding cutting performance, Rainbow Ultra demonstrated significantly higher cutting efficiency (*p* = 0.0168). Rainbow One was less flexible, as its maximum bending load (369 ± 34 gf) was higher than that of Rainbow Ultra (336 ± 14 gf) (*p* = 0.0161). Conversely, Rainbow One exhibited greater buckling resistance (339 ± 32 gf) than Rainbow Ultra (273 ± 24 gf) (*p* < 0.0001) (Table 2).

**TABLE 2:** Mean (standard deviation) results of time to fracture (in s), maximum torque (in N.cm), angle of rotation (in °), maximum bending load (in gf), buckling strength (in gf), and cutting ability (in gf) of tested instruments.

	Rainbow One	Rainbow Ultra	*p*
Time to fracture	79.1 ± 6.5	95.5 ± 7.4	*p* < 0.0001
Maximum torque	1.8 ± 0.2	1.4 ± 0.1	*p* = 0.0014
Angle of rotation	402 ± 50	485 ± 49	*p* = 0.0016
Maximum bending load	369 ± 34	336 ± 14	*p* = 0.0161
Buckling strength	339 ± 32	273 ± 24	*p* < 0.0001
Cutting ability	99.1 ± 13	84.6 ± 11	*p* = 0.0168

## 4 Discussion

This study provides a comprehensive evaluation of how dual heat treatment during manufacturing influences the mechanical performance of Rainbow One and Rainbow Ultra rotary NiTi instruments, considering key properties such as flexibility, cyclic fatigue resistance, torsional strength, bending, buckling, and cutting efficiency. Although the two systems share nearly identical geometrical designs, they demonstrated significant differences in mechanical behaviour, which were closely linked to distinct metallurgical characteristics. Minor dimensional variations of up to 40 μm, likely within standard manufacturing tolerances or arising from the multicoloured DLC coating, can be considered negligible and unlikely to affect mechanical outcomes. Importantly, these results demonstrate the decisive role of heat treatment protocols in defining the mechanical profile of NiTi instruments, revealing that even subtle variations in thermal processing can markedly alter performance. This finding underscores that the mechanical behaviour of NiTi instruments is strongly influenced by metallurgical modifications, providing new insights into how thermal processing can optimise instrument performance and supporting the rejection of the null hypothesis. It is also important to clarify the rationale for selecting Rainbow One and Rainbow Ultra for comparative analysis. To the best of our knowledge, they are the only commercially available NiTi systems in which single versus dual heat treatment is intentionally disclosed as the primary manufacturing distinction. Thus, the relevance of this comparison lies not in product‐specific considerations, but in the scientific value of examining how sequential heat treatments alone can modulate phase transformation behaviour and mechanical performance, an investigation that would not be feasible with most commercially available instruments.

According to the manufacturer, the main distinction between these two instruments lies in the heat treatment strategy. Rainbow One undergoes a single NiTi heat treatment after machining, whereas Rainbow Ultra is subjected to a heat treatment prior to machining and another following it. Pre‐machining heat treatment is intended to stabilise the bulk microstructure of the NiTi alloy before mechanical processing (Silva et al. 2025). By applying a controlled heating and cooling cycle, the alloy is softened, and residual stresses from wire production are largely relieved. This process may also facilitate the formation of a more homogeneous martensitic or R‐phase structure, enhancing ductility and machinability. In addition to optimising the alloy's thermomechanical properties, pre‐machining heat treatment aids precise and reproducible machining by minimising tool wear and surface irregularities. Nonetheless, subsequent machining can partially reintroduce localised stresses or induce work hardening, potentially affecting surface integrity and microstructural stability (Kuhn et al. 2001; Kwak et al. 2021; Wei et al. 2024).

In contrast, post‐machining heat treatment is designed to mitigate the residual effects induced by mechanical processing (Silva et al. 2025). Machining steps such as grinding, twisting, and milling commonly generate surface defects, work hardening, and residual stresses, which can act as stress concentrators and compromise fatigue strength (Kwak et al. 2021; Wei et al. 2024). Applying heat treatment after machining relieves these stresses by redistributing dislocations, refining grain boundaries, and stabilising the desired crystallographic arrangements (Kwak et al. 2021). Additionally, it promotes the formation of protective oxide layers and smooths surface irregularities (Silva et al. 2025), further enhancing fatigue resistance and reducing the risk of crack initiation. Collectively, the primary aim of post‐machining heat treatment is to restore structural equilibrium, minimise machining‐induced damage and ensure the final instrument exhibits improved flexibility, ductility and fatigue strength (Kuhn et al. 2001; Kwak et al. 2021; Wei et al. 2024).

When both heat treatments are applied sequentially, as in Rainbow Ultra—which undergoes a pre‐machining cycle (responsible for its distinctive black coloration) followed by a post‐machining cycle—the alloy likely benefits cumulatively from both stages. The pre‐machining treatment enhances machinability and promotes a more uniform internal structure, while the post‐machining cycle further relieves residual stresses and stabilises phase composition (Silva et al. 2025). This dual treatment may account for the higher phase transformation temperatures observed in Rainbow Ultra compared with Rainbow One (Table 1, Figure 3), including elevated R‐phase finish (Rf) and austenite finish (Af) values, which contribute to increased flexibility and fatigue resistance. The presence of a double austenitic peak in Rainbow Ultra, which is absent in Rainbow One, further suggests that the combined thermal history modifies the transformation pathway, producing a more complex but potentially advantageous microstructure. Nevertheless, other commercially available NiTi instruments also exhibit R‐phase at relatively high transformation temperatures (Martins et al. 2025), leaving it uncertain whether the observed increases in Rainbow Ultra are uniquely due to the dual heat‐treatment sequence or could be achieved with a single post‐machining cycle, and to what extent this would translate into mechanical performance differences.

Regarding mechanical performance, the superior cyclic fatigue resistance of Rainbow Ultra can be attributed to its higher R‐phase and austenite transformation temperatures, as confirmed by DSC data (Figure 3, Table 1). At the testing temperature (22°C), Rainbow Ultra retains a larger proportion of R‐phase (a martensitic state with enhanced flexibility) while Rainbow One may still contain residual austenite due to lower Rf values. The predominance of R‐phase in Rainbow Ultra reduces internal stress concentrations during rotation in curved canals, which may delay crack initiation and instrument fracture (Herold et al. 2007; Martins et al. 2022). Conversely, Rainbow One exhibited higher maximum torque in torsional testing, reflecting greater torsional strength. This corresponds to a higher dislocation density, lower Rs/Rf transformation temperatures, and elevated Ms/Mf values. Instruments with stiffer profiles can better resist torsional stress before plastic deformation or fracture, which may be advantageous in clinical scenarios such as taper lock in narrow canals (Silva et al. 2023). Analysis of the angle of rotation showed that Rainbow Ultra deformed more before fracture, indicating superior ductility. This is attributable to its higher R‐phase content, which accommodates greater plastic strain than the stiffer austenitic phase, potentially providing a safety buffer in complex canal anatomies or during unexpected locking events (Silva et al. 2023).

Bending tests revealed greater flexibility (lower bending strength) in Rainbow Ultra, consistent with its elevated Rf and Af values (Table 1, Figure 3), which favour a microstructure dominated by martensitic or R‐phase at the testing temperature. In contrast, Rainbow One likely retains residual internal stresses, resulting in higher stiffness. Enhanced flexibility, as observed in Rainbow Ultra, is considered clinically advantageous for preserving original canal curvature and reducing the risk of transportation (Martins et al. 2022). Buckling strength, which benefits from stiffer alloys, was higher in Rainbow One, reflecting residual stresses and lower transformation temperatures under test conditions. While this may aid tasks such as gutta‐percha removal or canal penetration (Lopes et al. 2012), higher stiffness can reduce flexural compliance in complex canal anatomies, where greater flexibility, like that of Rainbow Ultra, is preferred for safe and efficient shaping. Interestingly, Rainbow Ultra also exhibited superior cutting efficiency despite its overall flexibility. This seemingly counterintuitive result is likely due to microstructural differences arising from the distinct heat treatments, with a higher proportion of R‐phase or martensitic structures enhancing surface compliance and elastic recovery, thereby improving engagement of cutting blades with canal walls, in agreement with previous reports (Vasconcelos et al. 2018; Deari et al. 2020).

A comprehensive interpretation of the results must consider the clinical service temperature, which ranges from room temperature (~22°C) to slightly below body temperature, influenced by heat dissipation and irrigation during procedures (Atmeh et al. 2024). In this context, DSC‐derived transformation temperatures offer important insight into the expected behaviour of each instrument in clinical conditions. Rainbow One, with Rs around 31°C and Rf near 25°C, is likely to transition fully or almost fully to the austenitic phase under these temperatures, resulting in increased stiffness and reduced flexibility, which strongly affects mechanical performance (Elnaghy and Elsaka 2016; Dosanjh et al. 2017). Conversely, Rainbow Ultra, with higher Rs and Rf values (approximately 45°C and 35°C, respectively), is less likely to complete the austenite transformation, retaining a predominance of R‐phase or martensitic structures. This microstructural state maintains greater flexibility, ductility and fatigue resistance. Such phase‐dependent behaviour must be considered when extrapolating laboratory findings to clinical scenarios, as it directly impacts instrument performance during endodontic procedures (Silva et al. 2025). From a clinical perspective, the distinct transformation behaviours suggest that instruments retaining higher R‐phase content at operative temperatures may better navigate sharply curved canals with reduced risk of cyclic fatigue failure, whereas austenite‐dominant instruments may provide greater torsional robustness when negotiating narrow or highly constricted segments. These metallurgical distinctions, although requiring clinical validation, may ultimately guide more individualised instrument selection according to canal anatomy.

This study has some limitations that should be acknowledged. Experiments were conducted under standardised laboratory conditions, which cannot fully replicate the complex clinical environment where rotary instruments operate. Variables such as intracanal temperature fluctuations, canal anatomy, and operator‐dependent factors may affect instrument behaviour differently in vivo. Additionally, only a single instrument size and taper were evaluated, limiting direct extrapolation to other instruments or systems. Although the multimethod testing approach provides valuable complementary insights into design, mechanical, and metallurgical performance, further investigations using in situ or clinical protocols are necessary to confirm these findings under real operating conditions. Despite these limitations, the study offers several methodological strengths and novel contributions. While previous research has explored the effects of heat treatment and manufacturing processes on NiTi instruments, none have specifically tested how dual heat treatment during manufacturing influences their mechanical and metallurgical properties. By comparing two instruments with similar designs, but different heat treatments, this study isolates the impact of cumulative thermal processing on microstructure, phase transformation behaviour and mechanical performance. Furthermore, the multimethod experimental design, integrating geometric, metallurgical and mechanical analyses, strengthens the validity of the findings and offers a comprehensive understanding of the interplay between design, alloy structure and mechanical performance, representing a meaningful methodological advancement in endodontic materials research (Silva, Martins, and Versiani 2024).

Future investigations should aim to further clarify the effects of sequential heat treatments by experimentally isolating the phase transformations of the NiTi alloy. Designing instruments stabilised in distinct, well‐defined phase states would allow a more precise comparison of the mechanical behaviour associated with each thermal stage, namely pre‐ and post‐machining treatments. Achieving this, however, requires precise control over phase transformation pathways, making it technically challenging. Nevertheless, such investigations could yield deeper insights into the interplay between thermal processing, phase composition, and mechanical performance, ultimately guiding the optimization of NiTi instrument manufacturing strategies.

## 5 Conclusions

The application of dual heat treatment during manufacturing significantly influenced the mechanical performance of the tested rotary NiTi instruments. Rainbow Ultra, subjected to sequential heat treatments, exhibited higher flexibility, cyclic fatigue resistance and cutting efficiency, whereas Rainbow One demonstrated greater torsional strength and buckling resistance.

## Author Contributions

**Emmanuel João Nogueira Leal Silva:** conceptualization, analysis, experimental procedures, writing, review (lead). **Thyago Oliveira Cardoso:** analysis, experimental procedures, writing. **Jorge N. R. Martins:** conceptualization, analysis, experimental procedures, writing, review (lead). **Francisco Manuel Braz Fernandes:** analysis, experimental procedures. **Victor Talarico Leal Vieira:** analysis, experimental procedures. **Marco A. Versiani:** conceptualization, analysis, experimental procedures, writing, review, and editing (lead).

## Funding

This study was partially funded by FAPERJ and CNPq. FMBF acknowledges the funding of CENIMAT/i3N by national funds through the Fundação para a Ciência e a Tecnologia, I.P. (FCT), within the scope of Multiannual Financing of R&D Units, reference UIDB/50025/2020–2023.

## Ethics Statement

The authors have nothing to report.

## Conflicts of Interest

The authors declare no conflicts of interest.

## Supporting information


**Figure S1:** PRILE flowchart.

## Data Availability

The data that support the findings of this study are available from the corresponding author upon reasonable request.
