# Supplementary material for: Influence of Dual Heat Treatment on the Metallurgy and Mechanical Behaviour of Diamond‐Like Carbon–Coated NiTi Rotary Systems: A Multimethod Investigation
Source: Int Endod J. 2025 Dec 30;59(4):708–17. doi: 10.1111/iej.70089 (PMC12977936; doi:10.1111/iej.70089)
Supplement: Supplementary file 1 — Figure S1: PRILE flowchart. [file IEJ-59-708-s001.pdf]

PRILE 2021 Flowchart

The number of heat-treatments that one instrument receive during manufacturing remains largely unexplored. Understanding whether a dual-heat treatment can enhance mechanical properties is crucial for determining their clinical applicability.

To investigate how the application of dual heat treatment during the manufacturing process influences the mechanical performance (flexibility, cyclic fatigue resistance, torsional strength, bending, buckling, and cutting efficiency) of rotary nickel-titanium endodontic instruments.

Ethics Committe Approval protocol: NA

NiTi instruments with different heat-treatments

Rainbow One (n=58) and Rainbow Ultra (n=58)

Design (stereomicroscopy, scanning electron microscopy), metallurgy (energy-dispersive X-ray spectroscopy, differential scanning calorimetry), and mechanical performance (flexibility, cyclic fatigue resistance, torsional strength, bending, buckling, and cutting efficiency).

Stereomicroscopy, scanning electron microscopy, energy-dispersive X-ray spectroscopy, differential scanning calorimetry, mechanical tests.

Both instruments shared equivalent geometries. DSC revealed R-phase crystallographic structure at room temperature for both instruments; however, Rainbow Ultra exhibited higher phase transformation temperatures and a double austenitic transformation peak, compared with a single peak for Rainbow One. Mechanically, Rainbow Ultra demonstrated greater cyclic fatigue resistance, higher maximum rotation angle, and improved cutting efficiency ($p<0.05$). Rainbow One exhibited higher torque to failure, greater bending load, and higher buckling resistance, indicating stiffer behavior.

The application of dual heat treatment during manufacturing significantly influenced the mechanical performance of the tested rotary NiTi instruments.

This study was partially funded by XXX and XXX

The authors deny any conflicts of interest related to this study.

***From: Nagendrababu V, Murray PE, Ordinola-Zapata R, Peters OA, Rôças IN, Siqueira JF Jr, Priya E, Jayaraman J, Pulikkotil SJ, Camilleri J, Boutsioukis C, Rossi-Fedele G, Dummer PMH (2021) PRILE 2021 guidelines for reporting laboratory studies in Endodontology: a consensus-based development. *International Endodontic Journal* May 3. doi: 10.1111/iej.13542. https://onlinelibrary.wiley.com/doi/abs/10.1111/iej.13542.**

For further details visit: http://pride-endodonticguidelines.org/prile
